# A Conceptual Cortical Surface Atlas

**DOI:** 10.1371/journal.pone.0005693

**Published:** 2009-06-02

**Authors:** Dharmendra S. Modha

**Affiliations:** IBM Almaden Research Center, San Jose, California, United States of America; University of California Irvine, United States of America

## Abstract

Volumetric, slice-based, 3-D atlases are invaluable tools for understanding complex cortical convolutions. We present a simple scheme to convert a slice-based atlas to a conceptual surface atlas that is easier to visualize and understand. The key idea is to unfold each slice into a one-dimensional vector, and concatenate a succession of these vectors – while maintaining as much spatial contiguity as possible – into a 2-D matrix. We illustrate our methodology using a coronal slice-based atlas of the Rhesus Monkey cortex. The conceptual surface-based atlases provide a useful complement to slice-based atlases for the purposes of indexing and browsing.

## Introduction

The cortical surface is folded into a number of gyri and sulci creating complex topology that often baffles the mind. Over the last decade, various computational strategies have been developed to reconstruct and represent the spatial relationships of brain structures based on brain sections, either obtained from traditional anatomical experiments or via modern non-invasive approaches, such as magnetic resonance imaging. These approaches have led to volumetric, slice-based, 3-D stereotaxic atlases for completely capturing the cortical spatial complexity [1]–[3]. Several internet-enabled interactive atlases are also now available, see, for example, [4]. While these atlas are indispensable in their completeness, it is widely acknowledged that they are difficult to visualize [5]. Hence, complementary computerized surface-based atlases have gained in popularity [6]–[9]. Precise surface-based atlases are invaluable in individual registration with respect to the atlas. Since, a surface-based atlas cannot preserve all the information in a 3-D atlas, a vast amount of research has been invested in enhancing the surface-based atlases to counteract the missing details [10].

However, for neuro-anatomically-challenged lay people or for experts seeking only a bird's eye view, the precision and details can become impediments to achieving a simpler, conceptual overview of the cortical organization. Here, by sacrificing some precision, we are seeking a simple and intuitive surface-based atlas that is useful in providing a bird's eye view of the entire cortical surface while preserving as much information and details as possible.

While advocating “universal usability for information and communications technologies”, [11] stressed a need for “advanced strategies for satisfying first-time as well as intermittent and expert users”, and proposed encoding information in multiple layers where higher layers encode progressively more detailed information. Specifically, [11] put forth

“the idea of multi-layer interface designs that enable first-time and novice users to begin with a limited set of features at layer 1. They can remain at layer 1, then move up to higher layers when needed or when they have time to learn further features”.

In this context of multi-layer information presentation and visualization, we develop a simplified, conceptual cortical atlas that can be thought of as layer 1 that is suitable for novice and first-time users that are increasingly being drawn to neuroscience from a number of disciplines such as computer science, electrical engineering, mathematics, and so on. Further, our layer 1 atlas naturally links with the more detailed volumetric, slice-based atlas which can be subsequently consulted for more detailed information.

## Results

To illustrate concrete application of our methodology, we chose a slice-based atlas of the Rhesus monkey cortex created by respected neuroanatomists George Paxinos, Xu-Feng Huang, Michael Petrides, and Arthur W. Toga [12]. We chose this atlas for illustration since it was the “first comprehensive delineation of cortical and subcortical structures of any primate species” [12]. The atlas depicts the entire brain, consists of 151 photographs, 400 µm apart, and was produced with input from some of the most senior cortical neuroanatomists (Deepak Pandya and Lesley Ungerleider). To fully appreciate our results and methods, it will be helpful if the reader has ready access to [12].

We took each of the 151 slices and converted it into a one-dimensional column vector by enumerating the cortical areas in a ceratin linear order. We then concatenate a succession of these vectors – while maintaining as much spatial contiguity as possible – into a 2-D matrix which constitutes a conceptual surface atlas.

**Figure 1:**
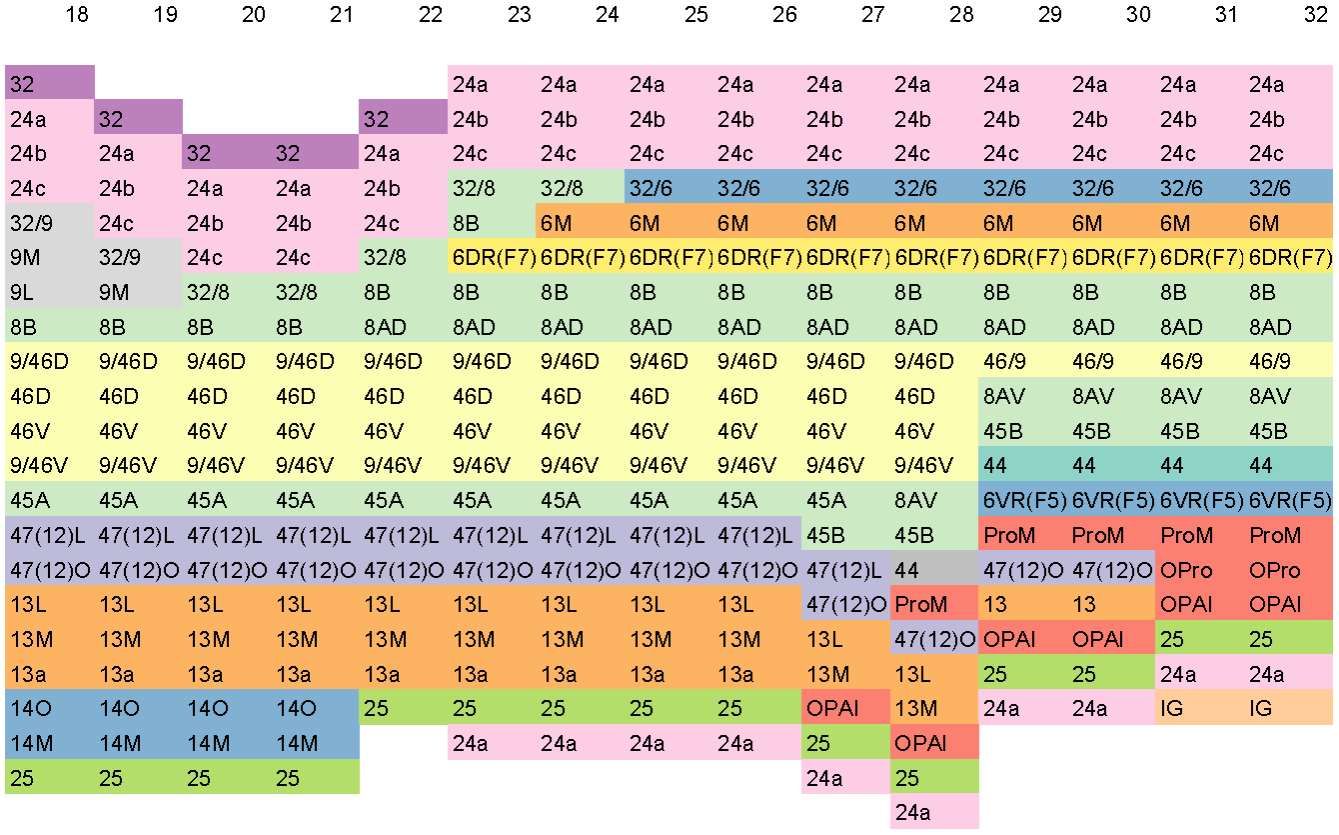
Fragment of the Conceptual Surface-based Atlas (Frontal Lobe). A fragment of the conceptual surface-based atlas derived from [12]. Columns are indexed from 18 through 32 corresponding to figures 18 through 32 in [12]. The fragment displays a portion of the frontal lobe. To enhance discrimination, different cortical areas are colored differently using 12-class, qualitative Set3 from [17].

Figure 1 shows a fragment of the conceptual surface-based atlas derived from [12] corresponding to a portion of the frontal lobe. Columns are indexed from 18 through 32 corresponding to figures 18 through 32 in [12].

**Figure 2:**
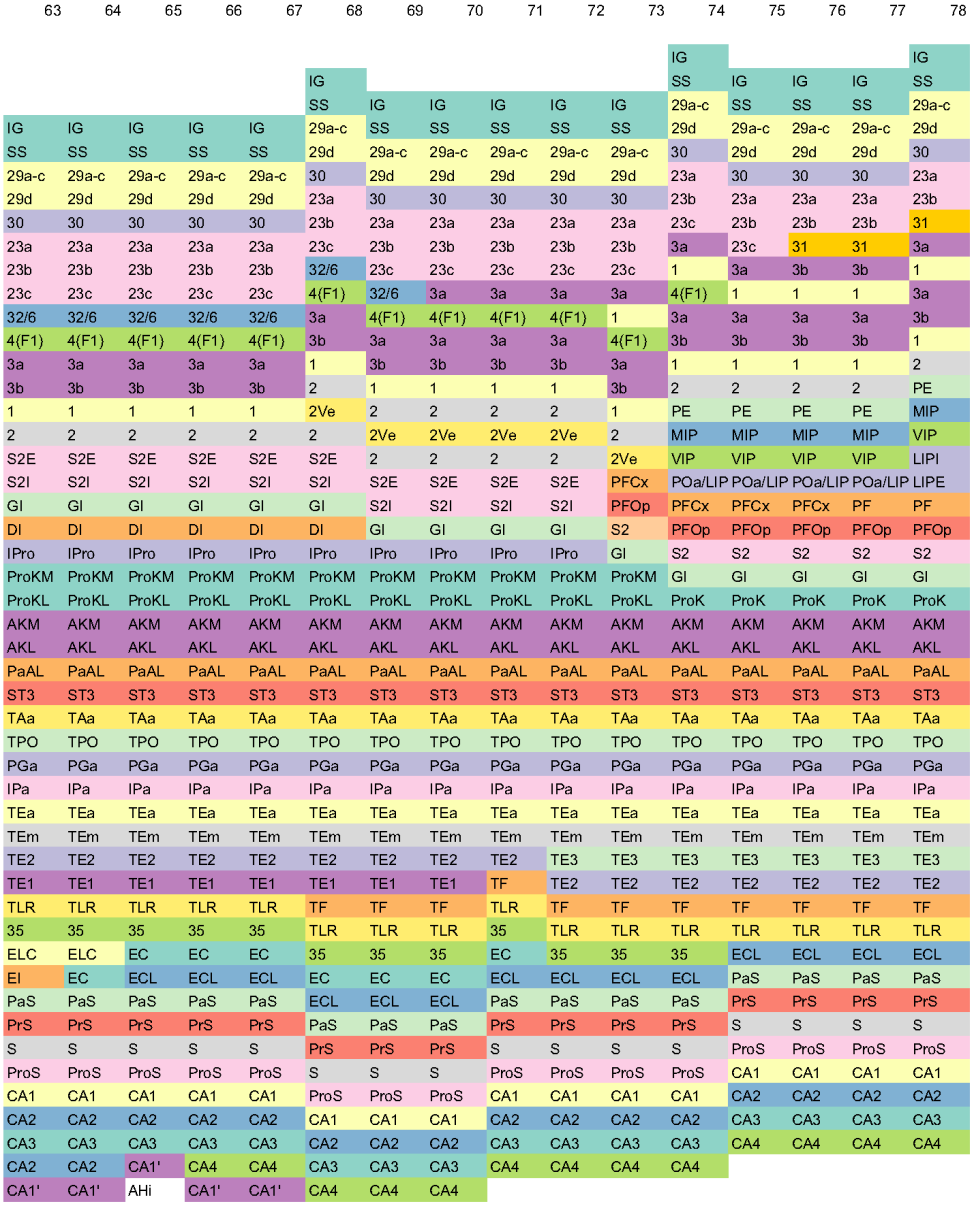
Fragment of the Conceptual Surface-based Atlas (Parietal and Temporal Lobes). A fragment of the conceptual surface-based atlas derived from [12]. Columns are indexed from 63 through 78 corresponding to figures 63 through 78 in [12]. The fragment displays portions of the parietal and temporal lobes. To enhance discrimination, different cortical areas are colored differently using 12-class, qualitative Set3 from [17].

Figure 2 shows a fragment of the conceptual surface-based atlas derived from [12] corresponding to portions of the parietal and temporal lobes. Columns are indexed from 63 through 78 corresponding to figures 63 through 78 in [12].

For the complete surface atlas including all 151 figures in [12], see Figure S1: Complete Conceptual Surface Atlas of the Rhesus Monkey Cortex. Figure S1 contains both Figures 1 and 2 as subsets. Further, in Figure S1, the rostral-to-caudal extent of each cortical area can be clearly seen. For example, the area TPO extends from slice 47 through 98. Further, relative extents of two or more areas can be easily gleaned, for example, CA1 and CA2 are co-extensive from slice 62 through 95.

In Figure S1, we have colored individual cortical areas. For a different perspective, see Figure S2 which is identical to Figure S1 except that we have colored larger groups (Frontal Lobe, Cingulate Cortex, Hippocampus+Parahippocampal Cortex, Insular Cortex, Temporal Lobe, Parietal Lobe, and Occipital Lobe).

## Discussion

Assuming that slices are cut at a uniform distance, our conceptual atlas is accurate horizontally (across slices) in that relative extents of cortical areas are preserved. However, vertical (within a single slice) extents are not preserved. To emphasize, the vertical thickness of each cortical area along a slice is the only missing information in the conceptual atlas. By sacrificing this detail, a tremendous simplification in representation is achieved. Specifically, the whole atlas can now be represented as a 2-D matrix that can be easily communicated via simple spreadsheets without the need for any detailed co-ordinates. If a co-ordinate system is developed along the surface of each slice, then our conceptual atlas can also be made accurate vertically, and, hence, can be converted into a precise surface-based atlas. Appealing to the multi-layer information presentation principle in [11], such a more precise atlas can serve as layer 2 while the detailed volumetric atlas would then serve as layer 3. In other words, easily digestable overview is presented first followed by details on-demand.

The illustrative atlas [12] used in this paper is based on coronal slices through the brain. Such an atlas naturally gives more prominence to subdivisions related to gyri and sulci that run rostral (anterior) to caudal (posterior), for example, superior temporal gyrus and sulcus. However, our methodology can also be easily applied to atlases based upon transversal or sagittal slices whereupon different gyri and sulci may be highlighted.

### Conclusions

The conceptual surface-based atlases provide an interesting and useful way to index, browse, visualize, and digest slice-based atlases and may prove to be a valuable complement to standard atlases such as [12]. Further, our scheme is easy and can be implemented in a matter of few days for any new slice-based atlas [13]–[16].

Finally, we have found that the conceptual atlas is very valuable in introducing neuro-anatomically-challenged lay people to the richness and beauty of the brain.

## Methods

We now present a scheme to convert a slice-based atlas into a conceptual visualization that was used to produce the results in Figures 1–2 and Figure S1, S2.

### Algorithm Components

Before we describe the precise work flow, we discuss two conceptual steps that are repeatedly used.

#### Enumerate

The main idea is to think of the cortical surface along each slice as a one-dimensional vector, and traverse it while enumerating the cortical areas that are encountered. The key variables in enumeration are (a) the starting area for enumeration and (b) the order in which the areas are enumerated. For example, for slice 23 in [12], area 24a serves as a natural starting point. Slice 23 can be converted into the following one-dimensional column or vertical vector (enumerated from top to bottom in a clock-wise fashion): 24a, 24b, 24c, 8/32, 8B, 6DR(F7), 8B, 8AD, 9/46D, 46D, 46V, 9/46V, 45A, 47(12)L, 47(12)O, 13L, 13M, 13a, 25, 24a. Each entry in this vector corresponds to a box in the 23rd column in Figure 1. For a full index of abbreviations, please see [12].

Some slices are extremely easy to enumerate. In particular, slices 23–32 and 46–89 have an unique starting point and an innate natural order from start to end. We term these slices as *anchors*. Each anchor slice has an unambiguous, absolute representation as a column vector. Anchor slices have the topology of a line segment, and geometry of a horseshoe. The only ambiguity in anchor slices is which of the two end points to pick as the starting point. We have consistently used the dorsal endpoint as the starting point.

The remaining slices have either no clearly defined start point or no clear order of enumeration. These *non-anchor* slices will be enumerated relative to the anchor slices.

#### Align

**Figure 3:**
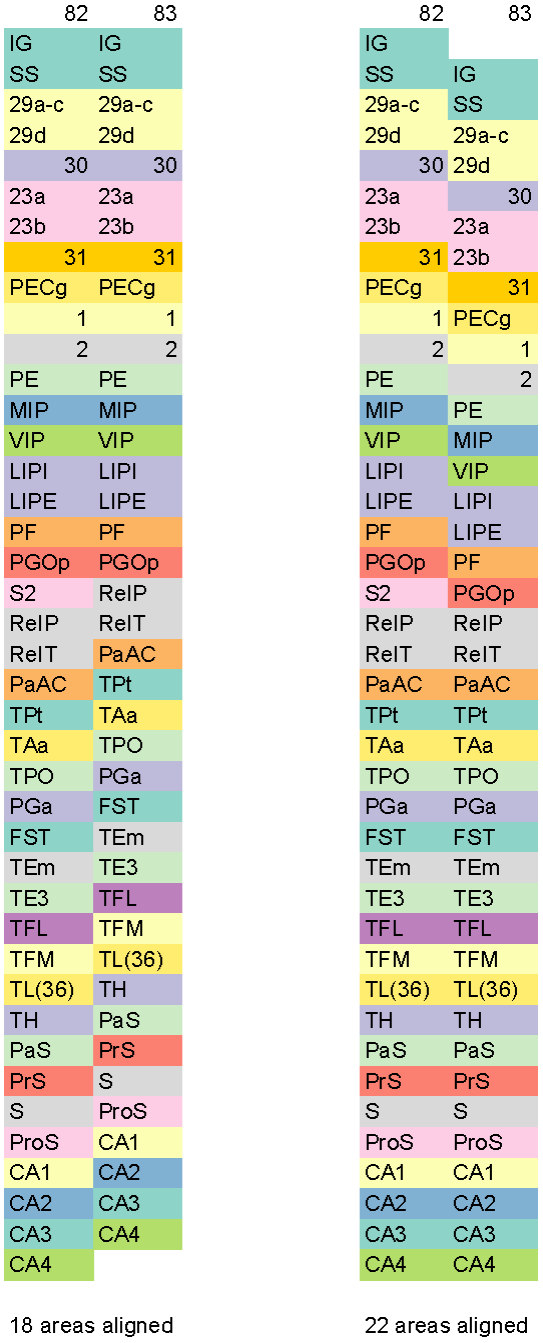
Better Alignment Leads to Lesser Visual Distortion. Two possible alignments for Columns 82 and 83 are shown. The alignment on left keeps 18 areas contiguous whereas the alignment on right keep 22 areas contiguous. Hence, we prefer the second alignment as it creates smallest visual distortion. To enhance discrimination, different cortical areas are colored differently using 12-class, qualitative Set3 from [17].

Let us suppose that we have enumerated two successive slices and created two column vectors. How should these column vectors be aligned with respect to each other? We would like to place them so that they visually appear maximally aligned. In other words, we would like to align the column vectors arising from successive slices so as to create minimal visual distortion by maintaining as much spatial contiguity between labeled cortical regions as possible. Less visual distortion leads to a surface-atlas that is easier to digest and study. As an example, in Figure 3, we show Columns 82 and 83. The first alignment shown on the left keeps 18 areas contiguous whereas the second alignment shown on the right keep 22 areas contiguous. Hence, we prefer the second alignment as it creates the smallest visual distortion via maximum alignment.

In addition to minimizing overall visual distortion, we will use the above alignment process to help select starting points and/or order of enumeration for non-anchor slices by relating them to neighboring anchor slices.

The enumerate and align steps are manual since a human decision is required to identify anchor slices, to determine how to enumerate non-anchor slices, and to seek the best alignment.

### Work Flow

We start by identifying anchor slices. Then, by using alignment with the anchor slices as a guide, we enumerate the remaining non-anchor slices.

#### Slices 23–32, 46–89

First, enumerate the anchor slices, namely, 23–32 and 46–89. Align anchor slices 23–32 so as to create minimal visual distortion, and ditto for slices 46–89. Figure 1 shows slices 23–32.

#### Slices 1–22

Slices 1 through 22 in frontal lobe have no logical beginning for enumeration step described above. They have the topology of a circle. For flattening them, a surgical cut is necessary.

We begin with slice 22. By using anchor slice 23 as a guide, for slice 22, we select a starting point, namely, area 32. Intuitively, this amounts to cutting through slice 22 at the boundary between areas 32 and 25 so as to endow the slice with a logical beginning for the purpose of enumerating. Our chosen cut is slightly dorsal to the rostral sulcus. Next, we enumerate the areas as: 32, 24a, 24b, 24c, 32/8, 8B, 8AD, 9/46D, 46D, 46V, 9/46V, 45A, 47(12)L, 47(12)O, 13L, 13M, 13a, and 25. Next, we align slice 22 to slice 23 so as to create minimal visual distortion. Now, slice 22 itself becomes an anchor, and we repeat the process for slice 21 by using slice 22 as a guide, and so on, until all slices 22-1 are cut, enumerated, and aligned. Figure 1 shows slices 18–32.

#### Slices 33–45

**Figure 4:**
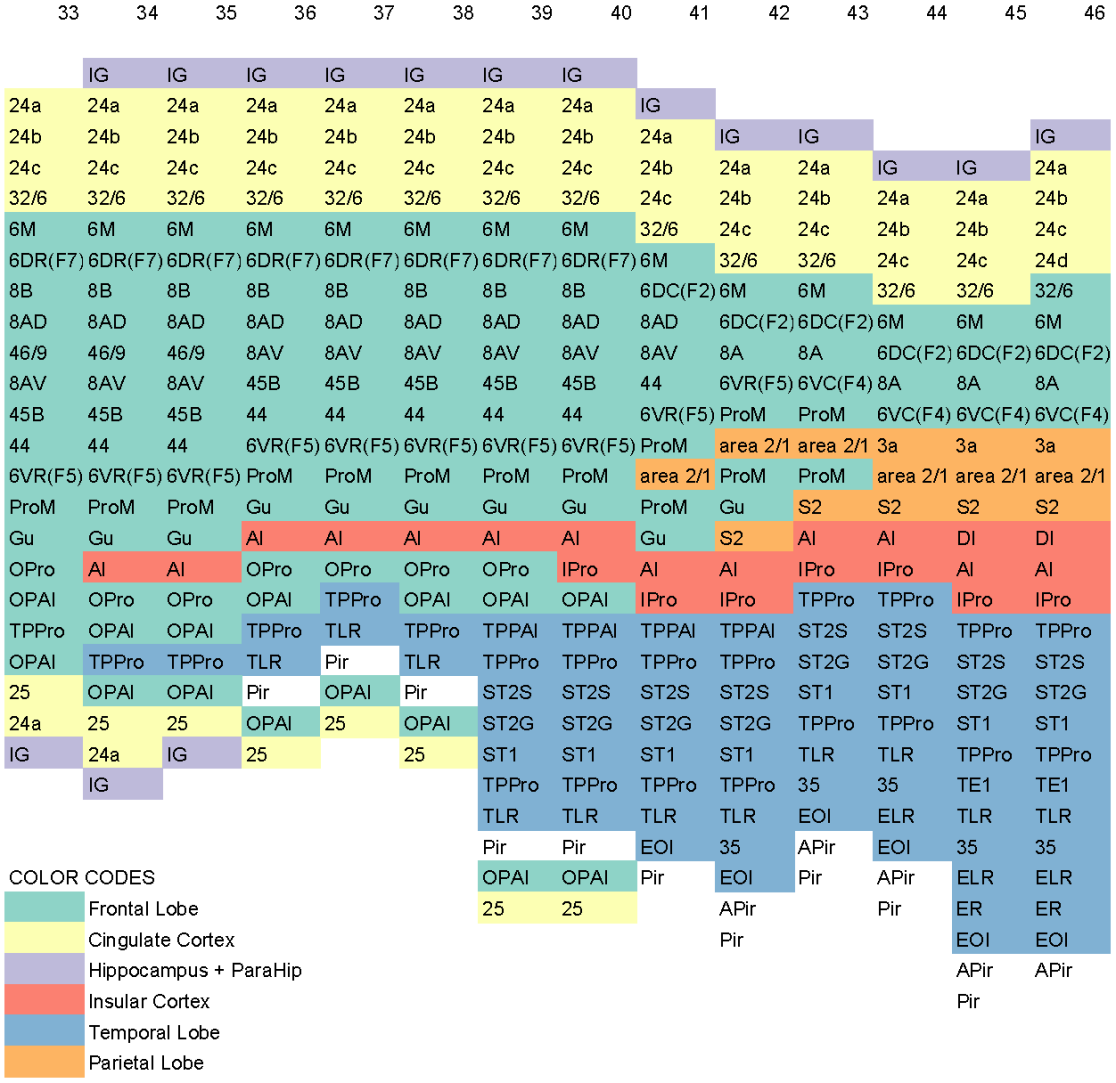
Columns 33–46 of the Conceptual Surface-based Atlas. A fragment of the conceptual surface-based atlas derived from [12]. Columns are indexed from 33 through 46 corresponding to figures 33 through 46 in [12]. Note the way in which temporal lobe and frontal lobe are shown as intertwined in columns 34–40. To enhance discrimination, different cortical areas are colored differently using 7-class, qualitative Set2 from [17].

Slices 33 through 45 are interesting because both the temporal lobe and the frontal lobe exist but as disjoint regions. For each slice, the starting point is easy to determine. The issue is how to interleave the cortical areas from two different lobes into a single ordering while minimizing the overall visual distortion. We use slice 46 on the right as an anchor slice and enumerate and align slices 45 through 33 in that order so as to create minimal visual distortion. No cut is necessary for these slices. Figure 4 shows slices 33–46. Observe how frontal lobe and temporal lobes are shown as intertwined in slices 34–40.

#### Slices 97–151

Slices 97–151 in occipital lobe have no logical beginning for enumeration step described above. For flattening them, a surgical cut is necessary. We cut slice 97 slightly ventral to superior parietal sulcus at the boundary of areas PGM and 23. Thus endowing it with an unique starting point, namely, area PGM. We now enumerate slice 97 as: PGM, PECg, PEC, MIP, VIP, LIPI, LIPE, PG, PGOp, ReI, TPt TPO, MST, MT(V5), FST, TEOm, TEO, V4V, V3V, V2, ProSt, 23, 30, 29d, 29a–c, and S. Finally, by using slice 97 as an anchor, we cut, enumerate, and align slices 98 through 151 in that order.

#### Slices 90–96

Slices 90–96 where the cingulate cortex meets the hippocampus are interesting.

In slices 90–92, cingulate gyrus (areas 23, 30, 29/29a) exists as a disjoint area. Otherwise, they have a clear starting point and a clear order of enumeration. For these slices, the disjoint regions are shown as such, and the remaining areas are shown with respect to slice 89 as anchor.

In slices 93–96, neither a clear starting point nor a clear order for enumeration exits. In this case, by using slice 97 on the right as an anchor, we cut, enumerate, and align slices 96 through 93 in that order.

**Figure 5:**
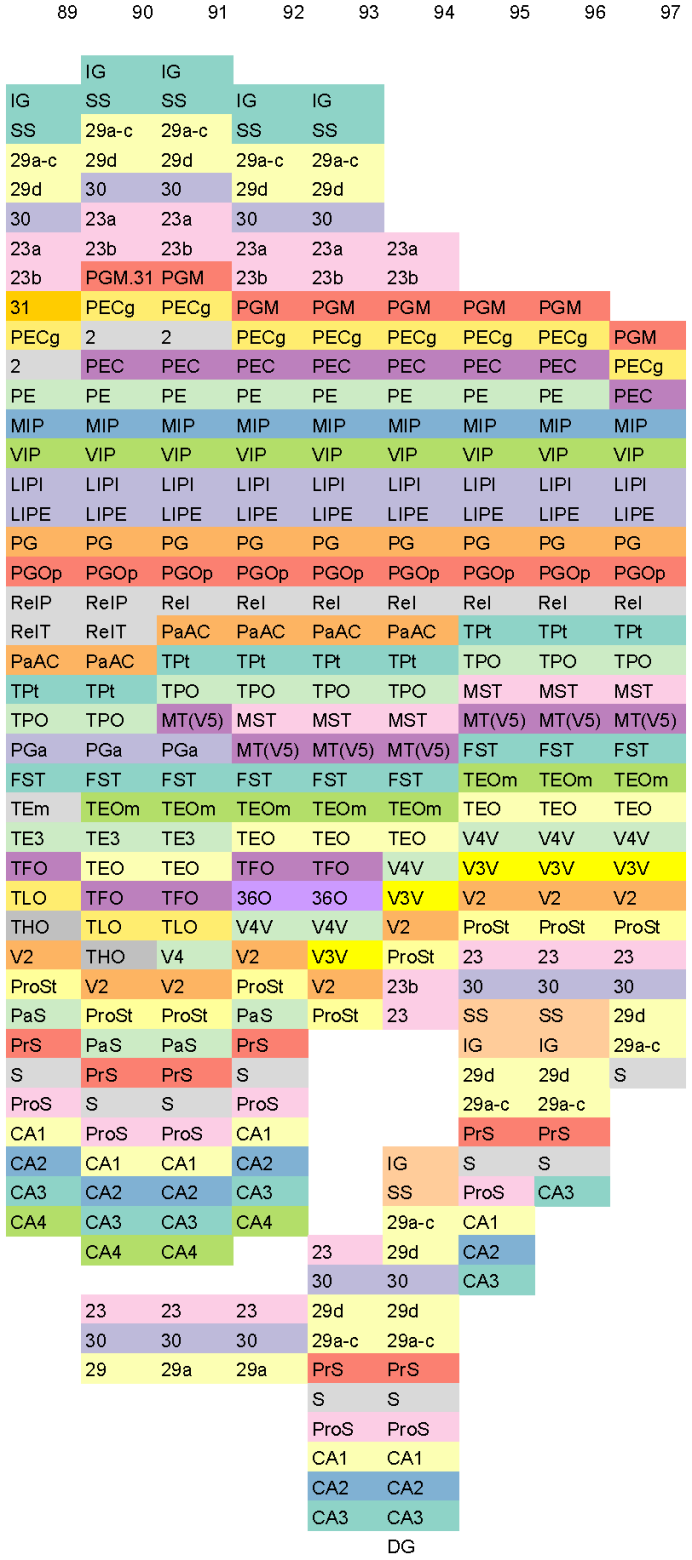
Columns 89–97 of the Conceptual Surface-based Atlas. A fragment of the conceptual surface-based atlas derived from [12]. Columns are indexed from 89 through 97 corresponding to figures 89 through 97 in [12]. The fragment displays slices where the cingulate cortex meets the hippocampus. To enhance discrimination, different cortical areas are colored differently using 12-class, qualitative Set3 from [17].

Figure 5 shows slices 89–97.

### Color

The final step is to color code different areas so as to enhance visual discrimination. In the conceptual atlas, some areas are shown as spatially contiguous although they are not, and some areas that are spatially contiguous are not shown to be so. This distortion is a by-product of the fact that we have no co-ordinate system in the vertical direction, that is, along each column vector. The drawback can be alleviated by introducing color coding to indicate spatial contiguity. Color visually links same or grouped entities in successive columns.

Application of color theory to cartography, is a well-studied art [17]. Accordingly, the colors in the figures are judiciously chosen. Specifically, for Figures 1, 2, 3, 5, and Figure S1, we have used 12-class, qualitative Set3 from [17]. For these figures, due to the limited number of available colors when compared to the number of areas, just as in a map, the same color is used multiple times for different areas that are not adjacent. For Figure 4 and Figure S2, we have used 7-class, qualitative Set2 from [17].

### Putting it Together

The entire conceptual cortical surface-based atlas is shown in Figures S1 and S2.

## Supporting Information

Figure S1 Complete Conceptual Surface-based Atlas. The figure shows the entire conceptual surface-based atlas derived from [12]. Figures 3 and 4 are subsets of this figure. Columns are indexed from 1 through 151 corresponding to figures 1 through 151 in [12]. To enhance discrimination, different cortical areas are colored differently using 12-class, qualitative Set3 from [17]. NOTE: The figure is meant to be printed as a poster.
(11.18 MB TIF)

Figure S2 Complete Conceptual Surface-based Atlas. This figure is identical to Figure S1, however, here we have colored larger regions such as the Frontal Lobe, Cingulate Cortex, Hippocampus+Para-Hippocampal Cortex, Insular Cortex, Temporal Lobe, Parietal Lobe, and Occipital Lobe in this file. To enhance discrimination, different cortical areas are colored differently using 7-class, qualitative Set2 from [17]. NOTE: The figure is meant to be printed as a poster.
(11.18 MB TIF)
