# Supplementary figures and images for: A Conceptual Cortical Surface Atlas
Source: PLoS One. 2009 Jun 2;4(6):e5693. doi: 10.1371/journal.pone.0005693 (PMC2685458; doi:10.1371/journal.pone.0005693)

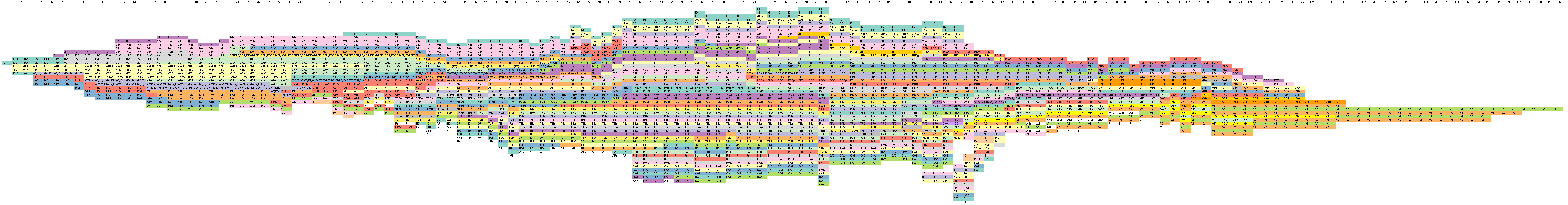

Supplement: Figure S1 — Complete Conceptual Surface-based Atlas. The figure shows the entire conceptual surface-based atlas derived from [12]. Figures 3 and 4 are subsets of this figure. Columns are indexed from 1 through 151 corresponding to figures 1 through 151 in [12]. To enhance discrimination, different cortical areas are colored differently using 12-class, qualitative Set3 from [17]. NOTE: The figure is meant to be printed as a poster. (11.18 MB TIF) [file pone.0005693.s001.tif]

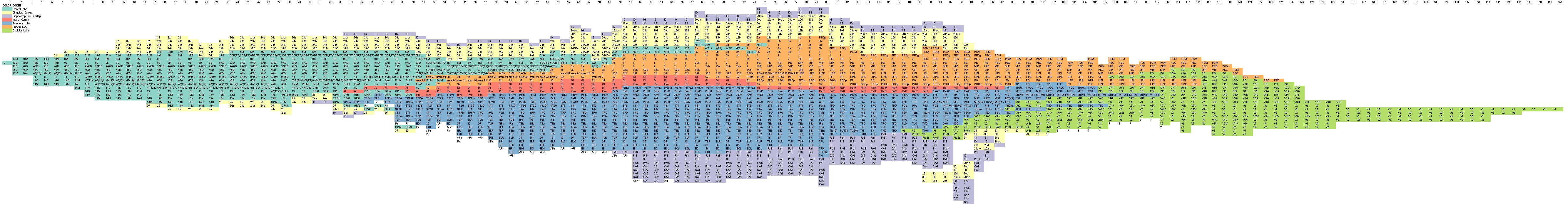

Supplement: Figure S2 — Complete Conceptual Surface-based Atlas. This figure is identical to Figure S1, however, here we have colored larger regions such as the Frontal Lobe, Cingulate Cortex, Hippocampus+Para-Hippocampal Cortex, Insular Cortex, Temporal Lobe, Parietal Lobe, and Occipital Lobe in this file. To enhance discrimination, different cortical areas are colored differently using 7-class, qualitative Set2 from [17]. NOTE: The figure is meant to be printed as a poster. (11.18 MB TIF) [file pone.0005693.s002.tif]
